# Spontaneous resolution of traumatic bronchial tear after thoracic crush injury

**DOI:** 10.1093/jscr/rjac627

**Published:** 2023-03-31

**Authors:** Erik Schwarze, Donald D Chang, Corbin J Cleary, Ilya Rakitin

**Affiliations:** Department of General Surgery, Henry Ford Hospital, Detroit, MI, USA; Department of General Surgery, Henry Ford Hospital, Detroit, MI, USA; Department of Thoracic Surgery, Henry Ford Hospital, Detroit, MI, USA; Department of Surgery, Division of Acute Care Surgery, Henry Ford Hospital, Detroit, MI, USA

**Keywords:** trauma, bronchial tear, tracheobronchial injury, blunt trauma

## Abstract

Traumatic bronchial tears are rare life-threatening injuries. Here, we report a 28-year old male who presented after sustaining a crush injury to his thoracic cavity, resulting in a spiral left mainstem bronchial tear secondary to high intraluminal pressure. While preparing for surgery, a preoperative bronchoscopy found that the bronchial tear had re-approximated and effectively sealed the laceration. No operative intervention was performed and the patient subsequently underwent a full recovery. While most bronchial tears undergo surgical intervention, our report describes the successful management of a bronchial tear injury with a non-operative approach and supportive care.

## INTRODUCTION

Tracheobronchial injuries due to blunt trauma are rare, life-threatening injuries associated with mortality rates as high as 80% [1, 2]. As such, the true incidence is hard to estimate, though prior studies have observed an incidence of around 0.4–2.8% of all traumatic injuries [3–5]. While no standardized management approach is in place, the widely accepted practice is that bronchial injuries resulting in complete ruptures should prompt surgical intervention [6]. Here, we present the case of a patient who suffered a full thickness tear of his left bronchial stem after a traumatic crush injury, who underwent successful non-operative management.

## CASE REPORT

**Figure 1:**
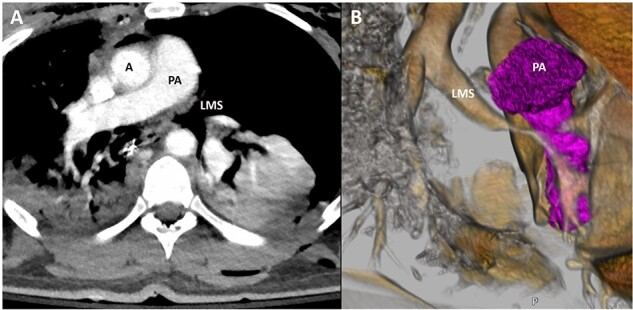
Representative image of axial slice of CT scan (**A**) and coronal 3D reconstruction (**B**) demonstrating narrowing of LMS secondary to bronchial injury; A, aorta; PA, pulmonary artery; LMS, left main stem.

A 28-year-old male hydraulic machinist suffered a mechanical crush injury primarily localized to his left thoracic cavity. He arrived at an outside hospital in respiratory distress with absent breath sounds noted within the left chest. He underwent emergent left-sided tube thoracostomy. Post-placement chest X-ray demonstrated persistent pneumothorax with midline shift, and a second chest tube was subsequently placed. Due to the nature of the injury with associated suspicion of a bronchial injury, the patient was intubated with a double lumen endotracheal tube and was placed on single right lung ventilation. Bronchoscopy at the outside hospital confirmed a left bronchial tear just distal to the carina. A computed tomography (CT) scan demonstrated bilateral pulmonary contusions, pneumomediastinum and a persistent large left hydropneumothorax (Fig. 1). Concurrent injuries also included a right radius and ulnar fracture. He was transferred to our Level 1 trauma, tertiary referral hospital’s surgical intensive care unit for escalation of care.

**Figure 2:**
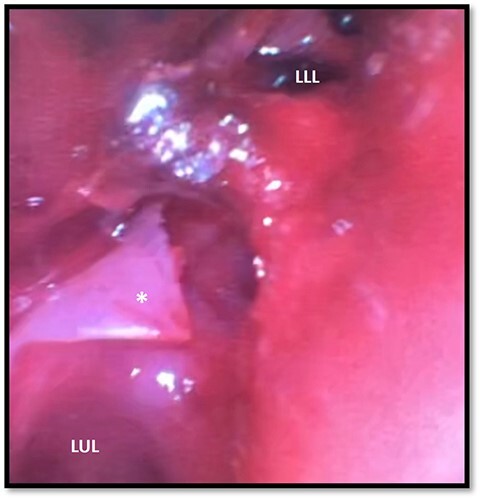
Bronchoscopy demonstrating left main stem bronchial tear (*) just proximal to the take between the LUL and LLL bronchi division; a hematoma can be seen obstructing the full view of the LLL; LUL, left upper lobe; LLL, left lower lobe.

**Figure 3:**
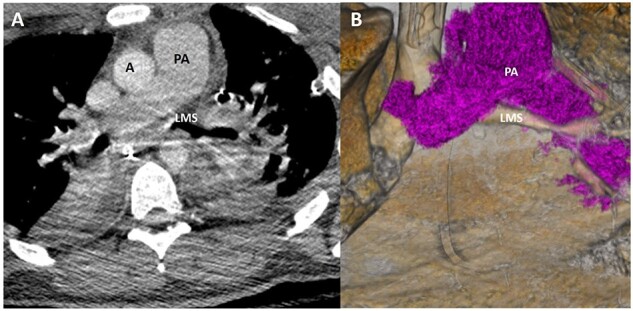
Representative image of axial slice of CT scan (**A**) and coronal 3D reconstruction (**B**) on HD 4 showed marked improvement in LMS patency.

The patient arrived hemodynamically stable and was intubated on single lung ventilation. Repeat bronchoscopy was performed at bedside, which demonstrated a full thickness tear in a spiral fashion spanning the medial to distal left mainstem bronchus with exposure of the pulmonary artery and communication with the mediastinum (Fig. 2). Surgical repair was planned for the following day with thoracic surgery. Prior to definitive repair, an intraoperative bronchoscopy was performed, which noted that the previously seen spiral left bronchial laceration had re-approximated into its original anatomical position, effectively sealing the lumen. Additionally, after double lung ventilation was restarted, there was no appreciable air leak. As such, the operative repair was deferred and the patient was managed with supportive care. A follow-up bronchoscopy was repeated on hospital day (HD) 4, which demonstrated that the left bronchial wall defect had remained closed with overlying intraluminal granulation tissue. Same day CT chest also demonstrated an interval decrease in pneumomediastinum and resolution of pneumothorax (Fig. 3). The patient underwent subsequent external fixation of his right radius and ulnar fractures. He was successfully weaned off of positive pressure ventilation on HD 11 and his chest tubes were subsequently removed. He was deemed to be medically stable for discharge on HD 15 and underwent outpatient stenting of his acquired bronchial stenosis.

## DISCUSSION

Bronchial disruptions are rare and are potentially dangerous injuries that are associated with high mortality rates [1].The prompt diagnosis and treatment of a bronchial tear is crucial to the successful management of the injury [6]. In the setting of blunt trauma, most bronchial tears occur within 2.5 cm of the carina [7], and tears in the right upper lobe bronchus have been observed to be more common compared to the left, an observation attributed to the protective effect of the aorta [8].

No standardized management approach exists for traumatic bronchial injuries, likely due to the rarity of the presentation. One study proposed stratifying surgical versus non-surgical management of bronchial tears based on lesion size, as they observed that the majority of patients with a bronchial injury of <2 cm recovered, though this study sample was small with only five patients and no control group [9]. Another study adopted a non-operative approach in 20 out of 33 traumatic bronchial injuries with favorable outcomes, although surgery was performed in any case with pneumomediastinum, pneumothorax or esophageal injuries [10]. Thus, in the setting of a compromised airway with pneumothorax or pneumomediastinum, the most widely accepted management approach is primary surgical repair through a standard thoracotomy. Injuries sustained within the left mainstem bronchus, as seen in our patient, are typically approached via a right thoracotomy [3].

Our patient suffered a left mainstem bronchial laceration most likely from increased intraluminal pressure after sustaining a hydraulic crush injury to the left thorax. Surgical repair was the correct indication. However, the spontaneous re-approximation of the spiral laceration with resolution of air leak on positive pressure ventilation was an unexpected finding. We postulated that the high intraluminal pressure resulted in spiral laceration of the cartilage but not of the mucosa, which lent itself to being spontaneously re-approximated into its former position when not under stress. As such, we opted for a non-operative approach with a low threshold to return to the operating room.

To our knowledge, this is the first case of traumatic bronchial tear to this degree of injury that spontaneously resolved without surgical intervention. Our case highlights the importance of a deliberate and systematic approach toward the surgical planning and management of these life-threatening injuries.
